# Wide Spectrum of Flecainide Toxicity: A Case Report and Literature Review

**DOI:** 10.7759/cureus.62923

**Published:** 2024-06-22

**Authors:** Anton Stolear, Maxim Dulgher, Ju Young Bae, Lila Kaminsky, Gini P Jeyashanmugaraja, Uzodinma Emerenini

**Affiliations:** 1 Cardiology, Yale University/Bridgeport Hospital, Bridgeport, USA; 2 Internal Medicine, Nuvance Health/Norwalk Hospital, Norwalk, USA; 3 Cardiac Electrophysiology, Yale New Haven Health/Bridgeport Hospital, Bridgeport, USA

**Keywords:** kidney injury, hallucinations, encephalopathy, loss of capture, atrial fibrillation, wide qrs, antiarrhythmic drugs, toxicity, flecainide

## Abstract

Flecainide toxicity is a rare but serious condition that can present with a wide range of clinical symptoms. We report the case of a 79-year-old female with paroxysmal atrial fibrillation on flecainide therapy who developed altered mental status, visual hallucinations, and bradycardia. Laboratory results revealed an acute kidney injury, which contributed to elevated flecainide levels. Discontinuation of flecainide led to a rapid resolution of symptoms and normalization of ECG findings. This case underscores the critical need for careful monitoring of renal function and potential drug interactions in patients receiving flecainide to prevent toxicity, highlighting the wide range of flecainide toxicity, including rare manifestations such as encephalopathy and visual hallucinations.

## Introduction

Flecainide is a class IC anti-arrhythmic drug (AAD) that can be used to treat supraventricular tachycardias such as atrial fibrillation (Afib). Its rate-dependent inhibition of fast sodium channels during phase zero results in slowed cardiac action potential upstroke and conduction of electrical impulses [1]. Flecainide has a narrow therapeutic index and is predominantly eliminated through renal excretion [2]. We present a case of wide complex bradycardia with loss of pacemaker capture and encephalopathy with hallucinations in the setting of flecainide toxicity and acute kidney injury (AKI).

## Case presentation

A 79-year-old female presented to the ED with recurrent diarrhea, followed by several days of altered mental status and visual hallucinations. Her past medical history included paroxysmal Afib treated with flecainide 150 mg twice daily, sinus node dysfunction status post-implantation of a Medtronic dual-chamber permanent pacemaker (PPM), and heart failure with preserved ejection fraction (HFpEF) managed with maintenance diuretic therapy. Emergency medical services found the patient to have an irregular rhythm and bradycardia with a heart rate of 20 beats per minute (bpm) and evidence of failure of ventricular capture of the PPM on the monitor. Upon hospital arrival, the patient had a systolic blood pressure of 70 mmHg and a heart rate of 40-60 bpm. She appeared dehydrated and was encephalopathic.

Pertinent laboratory findings revealed hyperkalemia: 5.8 mmol/L (normal range: 3.3-5.3 mmol/L), AKI with creatinine 1.01 mg/dL (baseline: 0.4-0.5 mg/dL), high-sensitivity troponin T 21 ng/L (repeat value: 16 ng/L, normal range: <12 ng/L), TSH 5.39 µIU/mL (normal range: 0.27-4.2 µIU/mL), and normal free T4 1.12 ng/dL (normal range: 0.8-1.7 ng/dL). The rest of the initial laboratory findings were within the normal range. The infectious workup was found to be unremarkable.

**Figure 1:**
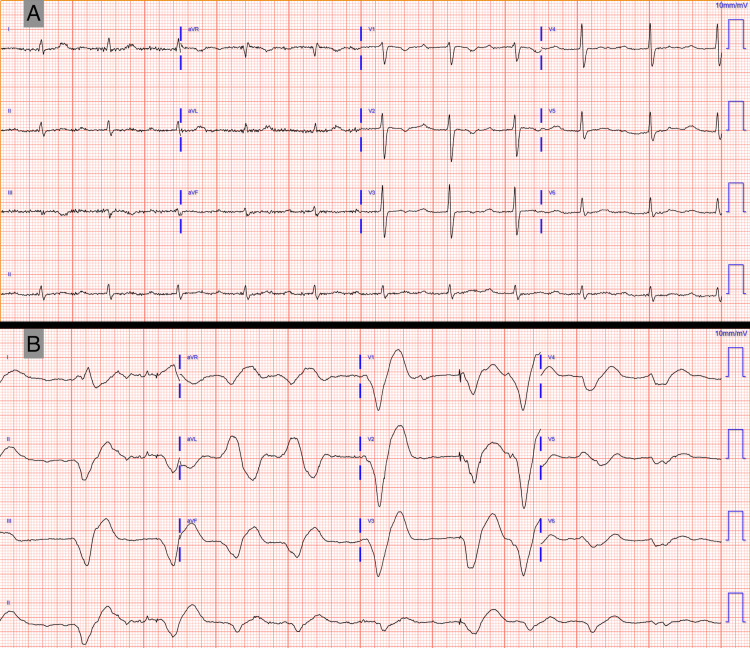
Comparison of baseline and presentation ECGs (A) Baseline ECG: sinus rhythm, heart rate 64 bpm, first-degree AV block (PR 344 ms), low voltage QRS in limb leads, nonspecific ST/T changes. (B) Presentation ECG: Afib with a QRS duration reaching 320 ms, ventricular paced second and sixth complexes. Afib, atrial fibrillation; AV, atrioventricular

The baseline ECG before this admission and the initial one on presentation were compared (Figure 1).

Norepinephrine and intravenous hydration were started for blood pressure support, and medical therapy for hyperkalemia was provided. A transthoracic echocardiogram revealed a normal left ventricular ejection fraction (LVEF) of 56% with paradoxical septal motion consistent with a wide complex QRS.

The cardiology service was consulted for the management of the wide complex rhythm. Of note, the patient has been taking flecainide for several years, and a PPM was implanted five months prior to the index admission. Based on the history of paroxysmal Afib on flecainide therapy, AKI, encephalopathy, and new ECG findings, flecainide toxicity was suspected, and the medication was discontinued.

Further workup included PPM interrogation, which revealed that the device has been set at VVI mode at a rate of 50 bpm with evidence of elevated right ventricular pacing threshold >2.5 V for the last four days (Table 1).

**Table 1:** PPM interrogation findings An elevated RV pacing threshold of >2.5 V was observed for four days preceding the index admission. PPM, permanent pacemaker; RV, right ventricular

Date	Time	Threshold	Amplitude (V)	Actual safety margin	Notes
March 30, 2024	1:00	>2.500 V	5	-	Abort – high threshold (>2.5 V)
March 29, 2024	1:00	>2.500 V	5	-	Abort – high threshold (>2.5 V)
March 28, 2024	1:00	>2.500 V	5	-	Abort – high threshold (>2.5 V)
March 27, 2024	1:00	>2.500 V	5	-	Abort – high threshold (>2.5 V)
March 26, 2024	1:00	2.375	4.75	2	Measurement OK
March 25, 2024	1:00	1.875	4.25	2.3	Measurement OK

**Figure 2:**
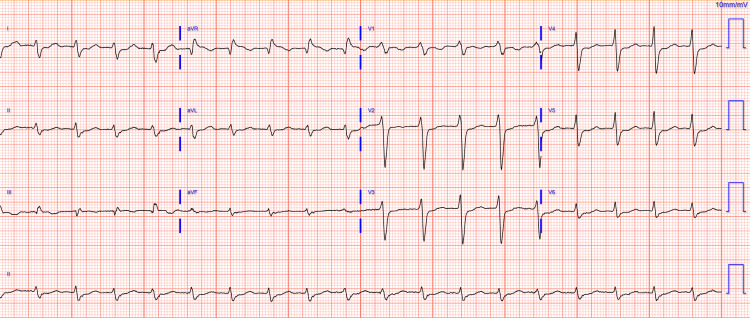
Repeat ECG on hospital day 3 Sinus tachycardia with a first-degree AV block. QRS duration 118 ms AV, atrioventricular

Over the 48 hours following the discontinuation of flecainide therapy, the patient’s mental status returned to baseline, hallucinations ceased, hemodynamic status stabilized, pressor support was discontinued, AKI resolved, and ECG findings improved (Figure 2).

The patient was discharged home on hospital day 5 in a stable condition. The flecainide level was obtained at the time of admission, and the result, which was received 16 days later, was found to be 2.96 mcg/mL (normal range: 0.2-0.99 mcg/mL). A close follow-up appointment was scheduled at the electrophysiology clinic one week after discharge to monitor clinical progress post-hospitalization.

## Discussion

Flecainide acetate belongs to the Vaughan-Williams class IC arrhythmic drugs (AADs). Its development process began in 1966 with the intent of creating a new anesthetic agent, and it was synthesized in 1972. Flecainide received US FDA approval in 1985 for the management of sustained ventricular tachycardia [2]. Safety data from 1980 and 1985 revealed that flecainide was linked to an elevated occurrence of proarrhythmic events, particularly in patients with underlying cardiac disease. To address this observation, a randomized clinical trial called the Cardiac Arrhythmia Suppression Trial (CAST) was initiated in 1987, which studied the use of flecainide among four AADs compared to placebo. This trial aimed to evaluate whether suppressing premature ventricular contractions (PVCs) could prevent sudden cardiac death in patients with frequent PVCs following a recent acute myocardial infarction (AMI). However, during a 1989 interim analysis, two tested AADs, encainide and flecainide, showed increased mortality compared to placebo. Consequently, their use was halted, leading to protocol revisions and the initiation of CAST II, which compared ethmozine to a placebo. However, CAST II was also terminated in 1992 due to higher mortality and nonfatal cardiac arrests in the ethmozine group. Despite successful PVC suppression, proarrhythmic fatalities were seen with similar arrhythmia and ischemic event rates but higher lethality in the active drug groups. The FDA added a black box warning to flecainide’s label, contraindicating its use in structural heart disease [1]. As the suppression hypothesis was disproved, the conventional practice of employing AADs to suppress asymptomatic arrhythmias in the setting of AMI changed.

Flecainide is rapidly and extensively absorbed after oral intake, peaking in plasma levels within two to four hours. While its half-life ranges from seven to 23 hours in patients with normal kidney function, in patients with end-stage renal disease, clearance is considerably reduced, prolonging the half-life up to 58 hours. Flecainide metabolism primarily involves the enzymatic activity of CYP2D6 and CYP1A2. This emphasizes the crucial importance of considering drug interactions that utilize the same metabolite pathway as flecainide. Approximately 86% of an oral dose of flecainide is excreted in the urine [2]. Renal elimination highlights the need for monitoring and dosage adjustments in patients with renal impairment.

**Figure 3:**
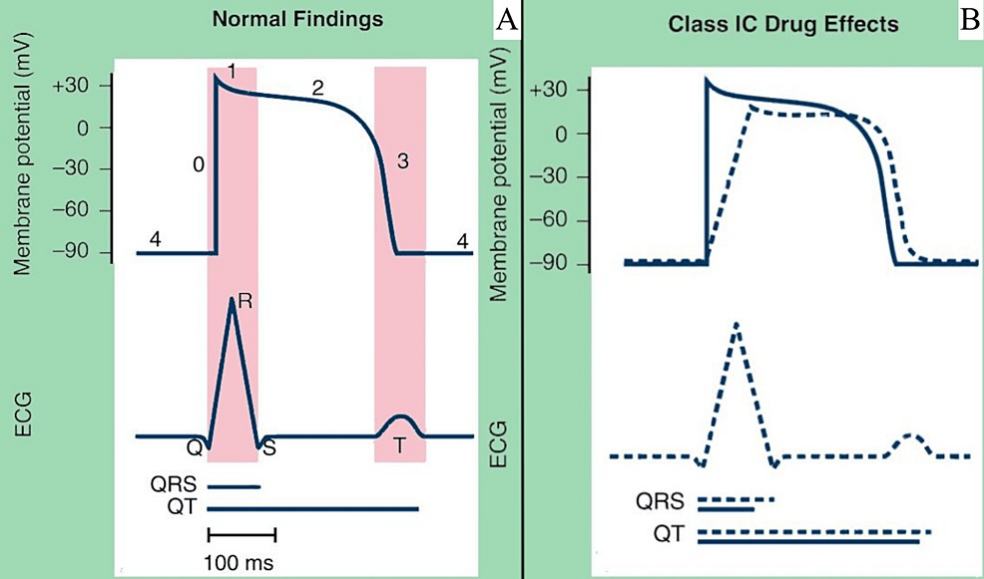
Cardiomyocyte action potential (A) Normal cardiomyocyte action potential. (B) Effect of class IC AADs on cardiomyocyte action potential. AAD, arrhythmic drug

Flecainide’s mechanism of action involves rate-dependent inhibition of phase 0 of the fast sodium channels, exhibiting a high affinity for open-state sodium channels. This action results in the slowing of the upstroke of the cardiac action potential, thereby reducing the conduction velocity of electrical impulses, predominantly affecting the His-Purkinje system and ventricular myocardium (Figure 3). Consequently, the anti-arrhythmic efficacy of flecainide is closely associated with changes in the QRS duration [3]. The phenomenon of slow unbinding kinetics from these channels during diastole has been elucidated, contributing to the prolonged recovery time during cardiac diastole and the extension of the refractory period. Flecainide additionally inhibits the opening of delayed rectifier potassium channels (Ikr), leading to the prolongation of action potential duration across ventricular and atrial muscle fibers [4]. Lastly, flecainide also blocks ryanodine receptor (RyR2) opening, leading to a reduction in spontaneous sarcoplasmic reticulum calcium release. This mechanism explains its therapeutic effect in the management of catecholaminergic polymorphic ventricular tachycardia [5].

The effect of flecainide on conduction velocity has been investigated at an electrophysiologic level. It slows both intra-atrial and atrioventricular (AV) nodal conduction. The drug lengthens the AH interval, reflecting AV nodal conduction time, by 15-22%. Similarly, it extends the HV interval, representing conduction from the His bundle to the ventricular myocardium, by 25-50% [6]. Additionally, as observed in our case, patients with implanted cardiac devices receiving flecainide therapy have exhibited documented increases in pacing thresholds attributable to heightened refractoriness [7]. Flecainide should be avoided in patients with existing poor thresholds or nonprogrammable PPM unless suitable pacing rescue is available. The pacing threshold in patients with PPM receiving flecainide therapy should be determined at regular intervals.

The proarrhythmic characteristics of class IC AADs have been extensively documented. These agents have the potential to convert Afib into atrial flutter with a relatively slow flutter rate, which may permit 1:1 AV conduction. This can lead to rapid tachycardia, which is characterized by a ventricular rate exceeding 200 bpm [8]. Hence, the administration of AV blocking agents before initiating class IC anti-arrhythmic therapy is recommended to mitigate the risk of rapid AV conduction and reduce the likelihood of exacerbating malignant ventricular arrhythmias. The proarrhythmic effect of class IC AADs occurs at a rate of approximately 3.5-5% [9]. Ventricular tachycardias induced by the proarrhythmic effects of class IC therapy appear to be infrequent in patients without underlying structural heart disease, coronary artery disease, or significant electrolyte abnormalities.

In addition to its impact on the intracardiac conduction system, flecainide also exerts a negative inotropic effect. Consequently, it is contraindicated in patients with underlying congestive heart failure with reduced ejection fraction due to its potential to further diminish stroke volume index and LVEF [10].

Flecainide finds utility in both the acute pharmacologic cardioversion of Afib and as a long-term therapeutic option to prevent its recurrence. The efficacy and safety of its prolonged use for this purpose have been subject to extensive investigation. However, owing to its narrow therapeutic index, with recommended plasma concentrations falling within the range of 0.2-1.0 mcg/mL, even slight variations in dosage or blood concentration may precipitate severe therapeutic shortcomings or life-threatening adverse reactions [3].

Flecainide toxicity is rare, but mortality is high (up to 22.5%), so awareness, early recognition, and treatment are paramount. Flecainide toxicity can lead to the rapid onset of symptoms, with nausea and vomiting being the most reported in the first 30 minutes after an overdose. Within two hours of ingestion, serious cardiac conduction disturbances may occur, leading to hypotension, shock, and cardiac arrest. These adverse effects stem from excess cardiac sodium channel blockade, resulting in delayed conduction, negative inotropy, various degrees of AV block, and dysrhythmias including bradyarrhythmia, ventricular tachycardia/fibrillation, and asystole [11]. As a result of the electrophysiological actions of flecainide on the cardiac cycle, surface ECG findings typically demonstrate prolongation of PR, QRS, and QT intervals [3]. Flecainide-induced neurotoxicity symptoms are rare and can include acute encephalopathy, hallucinations, and seizures. Visual hallucinations are an exceptionally rare manifestation of flecainide-induced neurotoxicity, with only a few reported cases to date [12]. The diagnosis of flecainide toxicity is based on clinical suspicion and ECG findings, as the serum drug level may take days to weeks to show results. In our case, it took 16 days for the results to become available.

The drug has a large volume of distribution, low protein binding, and a long elimination half-life, complicating the treatment of severe overdoses. Treatment of flecainide toxicity involves blocking its effects on the heart, correcting aggravating conditions for arrhythmias, such as electrolyte disturbance, hypotension, or hypoxia, and avoiding drugs with sodium channel blocking effects.

Sodium bicarbonate is used to decrease sodium channel blockade, and response to treatment is monitored with frequent ECGs, continuous cardiac monitoring, and blood gas analysis targeting a pH of 7.45 to 7.55. Conventional anti-arrhythmics may be ineffective or worsen flecainide-induced dysrhythmias by exacerbating sodium channel blockade [13]. Hemodialysis, peritoneal dialysis, and hemofiltration are not effective in eliminating flecainide. Because of the large volume of distribution, plasma exchange is also unsuccessful [14]. Intravenous lipid emulsion therapy has been proposed to decrease serum concentrations. The suggested mechanism revolves around establishing a lipid reservoir that attracts and isolates lipophilic medications in the bloodstream, thereby diverting them from their binding sites on cardiac myocytes. Emergency extracorporeal circulatory support may be necessary to restore vital organ perfusion for drug metabolism and elimination [15].

## Conclusions

This case highlights the critical importance of awareness and prompt recognition of the electrocardiographic and clinical manifestations of flecainide toxicity. Early identification allows for timely management, ultimately improving patient outcomes. In our case, flecainide toxicity was likely related to an AKI secondary to volume depletion from recurrent diarrhea and ongoing diuretic therapy for the management of HFpEF. This underscores the importance of considering renal function and fluid balance when prescribing medications with narrow therapeutic indices, such as flecainide, especially in patients at risk of volume depletion and AKI. Close attention should be paid to drug interactions, as they can impact flecainide levels and precipitate toxicity. Additionally, patients with implanted cardiac rhythm devices receiving flecainide therapy may encounter elevated pacing thresholds and failure to capture due to increased refractoriness. The pacing threshold in patients with PPM receiving flecainide therapy should be determined at regular intervals.
